# Clive Bell’s “Significant Form” and the neurobiology of aesthetics

**DOI:** 10.3389/fnhum.2013.00730

**Published:** 2013-11-12

**Authors:** Semir Zeki

**Affiliations:** Wellcome Laboratory of Neurobiology, Department of Cell and Developmental Biology, University College LondonLondon, UK

**Keywords:** neuroesthetics, significant configuration, visual brain, beauty, visual motion, faces, bodies

## Abstract

Though first published almost one century ago, and though its premise has been disputed, Clive Bell’s essay on aesthetics in his book *Art* still provides fertile ground for discussing problems in aesthetics, especially as they relate to neuroesthetics. In this essay, I begin with a brief account of Bell’s ideas on aesthetics, and describe how they focus on problems of importance to neuroesthetics. I also examine where his premise falls short, and where it provides significant insights, from a neuroesthetic and general neurobiological point of view.

## INTRODUCTION

In his book *Art* (Bell, 1914), Clive Bell (1881–1964), the English art critic, “tried to develop a complete theory of visual art…in the light of which the history of art from paleolithic days to the present becomes intelligible,” a theory which would give “a definite meaning” to terms such as “good drawing,” “magnificent design,” “unfelt,” “ill-organized.” The basis of Bell’s theory is that, “…there is a particular kind of emotion provoked by works of visual art…the aesthetic emotion”; this same emotion is provoked “by every kind of visual art” and works as diverse as “St Sophia and the windows at Chartres, Mexican sculpture, a Persian bowl, Chinese carpets, Giotto’s frescoes at Padua, and the masterpieces of Poussin, Piero della Francesca, and Cézanne” because “either all works of visual art have some common quality, or when we speak of ‘works of art’ we gibber.” Although his list includes architecture, paintings, frescoes, and *objets d’art*, it excludes musical or other works. He nevertheless does not consider his “aesthetic emotion” to be aroused solely by visual art. Though claiming not to be musical, he writes of music that, “…at moments I do appreciate music as pure musical form, as sounds combined according to the laws of a *mysterious necessity*, as pure art with a tremendous significance of its own” (my emphasis). For him, there is as well a strong relation between mathematical and artistic beauty, for the mathematician feels an emotion for his speculations which “springs… from the heart of an abstract science. *I wonder, sometimes, whether the appreciators of art and of mathematical solutions are not even more closely allied*” (my emphasis and ellipsis). This statement is not very different from that of Bertrand Russell (1917) in his book *Mysticism and Logic*, that “Mathematics, rightly viewed, possesses not only truth, but supreme beauty…. The true spirit of delight, the exaltation, the sense of being more than Man, which is the touchstone of the highest excellence, is to be found in mathematics as surely as poetry” (my ellipsis). Hence when Bell writes that, “…if we can discover some quality common and peculiar to all the objects that provoke [the aesthetic emotion], we shall have solved what I take to be the central problem of aesthetics” he is, I take it, not referring to visual art alone but to everything that is capable of arousing the “aesthetic emotion”, even if in his book he writes almost exclusively about visual art. His use of the term “mysterious necessity” is interesting, for it raises the question “necessity for what?” Implicit in my interpretation below is that the “necessity” is one for preferential or privileged activation of specialized areas of the brain.

The “common quality” that Bell refers to is, to a large extent, “a purely subjective business” because “any system of aesthetics which pretends to be based on some objective truth is so palpably ridiculous as not to be worth discussing” and because “All systems of aesthetics must be based on personal experience – that is to say, they must be subjective.” If it is subjective, as he says, but also independent of culture and learning, then it is probably worth asking whether it lies in some common neural organization that leads to personal (subjective) experiences, in the form of an “aesthetic emotion,” common to all humans. Thus one challenge raised by Bell’s formulation is to enquire whether there is any common quality in terms of brain activity that underlies the “aesthetic emotion” that he speaks of. But his reference to some quality “common and peculiar to all objects,” together with his list and description, nevertheless shows that he was also looking for some quality or characteristic in objects themselves, even though he professes to make of the “aesthetic emotion” a purely subjective business. Thus another neurobiological challenge in Bell’s formulations lies in trying to account for the extent to which the experience of beauty in such diverse sources as mathematics, visual art and music can be accounted for by objective qualities which are preferred by all humans because these diverse sources activate similar neural configurations common to the organization of our nervous systems and hence independent of culture and education.

Although not concerned with neurobiology, Bell’s formulations thus raise interesting points about the neural systems that underlie the experience of beauty and, in this somewhat speculative essay, I try to frame the neurobiological insights that his formulations give for an experimental approach to the study of beauty. Critical to Bell’s formulations is his concept of “*Significant Form*,” by which he means certain combinations of lines and colors which arouse the “aesthetic emotion.” This is a useful nucleus from which to build a more comprehensive view, of significant *configurations* that go beyond form and color and include many other visual attributes which can arouse the aesthetic emotion.

## THE COMMON FACTOR IN THE EXPERIENCE OF BEAUTY

The first neurobiological challenge in Bell’s theory, then, is to seek the common (subjective) factor in all that is experienced as beautiful, by transforming his question about what common property all works that arouse the “aesthetic emotion” have, into: is there a common mechanism in the brain that underlies the experience of beauty, regardless of source and regardless also of culture and experience?

**FIGURE 1:**
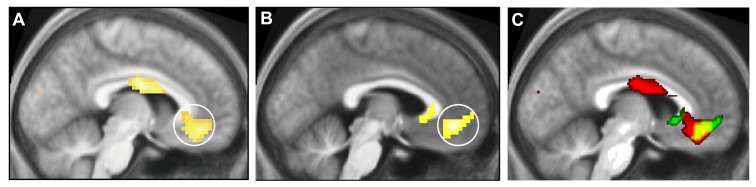
**Cortical activation correlating with the experience of beauty.** Brain activity obtained through **(A)** the contrast *Visually Beautiful > Visually Ugly*, **(B)** the contrast *Musically Beautiful > Musically Ugly*. Panel **(C)** shows the results of a conjunction analysis to reveal the areas of overlap in the activity produced in the medial orbito-frontal cortex (mOFC; circled in **A** and **B**) when subjects experienced visual beauty (red) and musical beauty (green). The zone of overlap is shown in yellow. From Ishizu and Zeki (2011).

Experiments which aim to determine the activity in the brain that correlates with the experience of beauty have repeatedly shown that there is one area, located interestingly in a part of the emotional brain known as the medial orbito-frontal cortex (mOFC) of the frontal lobes (**Figure 1**; see Ishizu and Zeki, 2011 for a review). This area is consistently active when subjects, irrespective of race or culture, report having had an experience of the beautiful, regardless of whether the source is visual, musical, or mathematical (Ishizu and Zeki, 2011; Zeki et al., unpublished); or whether, when visual, its source is in portrait, landscape or abstract painting and, when musical, its source is in symphonic works or jazz.

This implies that there is some degree of abstraction for beauty in the brain. This is not to say that this area alone is active during such experiences, or that there is “a beauty spot” in the brain; nor does it imply that the aesthetic emotion is aroused because of activity in this area alone. Instead, where the experience is of visual beauty, the input to the mOFC is through the visual brain; where it is of musical beauty, the input is through the auditory brain. Hence these areas, together with other regions of the brain such as the sub-cortical caudate nucleus for visual stimuli, are co-active with the mOFC. But it is significant that the mOFC is the only common area in which activity correlates with the experience of beauty derived from different sources. At present, it seems to be the only common (neural) property to all that is experienced as beautiful.

## EXPERIENCE OF THE PLEASANT, OF REWARD AND OF THE BEAUTIFUL

Reward and pleasure have commonly been written about in the philosophy of aesthetics, in relation to the experience of beauty (Graham, 2000). It is therefore interesting to note that the mOFC has also been found to be consistently active in the experience of reward and pleasure. This raises a very interesting problem, so far un-addressed: what, if any, is the difference in brain activity that correlates with the experience of different kinds of hedonic experience – the experience of beauty on the one hand and the experience of something as being pleasant or rewarding on the other. The mOFC is a large expanse of cortex with several cytoarchitectonic sub-divisions (see Kringelbach, 2005 for a review) and the available evidence suggests that not all kinds of reward and pleasure activate field A1 of mOFC. While it seems very likely that preference for drinks (McClure et al., 2004) correlates with activity in the same part of mOFC (field A1) as does the experience of abstract (O’Doherty et al., 2001) and predictive reward (Gottfried et al., 2003) or preference for certain kinetic patterns (Zeki and Stutters, 2013), it would appear that the hedonic experience of food correlates with a more lateral part of orbito-frontal cortex than A1 of mOFC (Kringelbach et al., 2003) while the experience of erotic pleasure appears to correlate with a region dorsal to field A1 (Sescousse et al., 2010). These are approximations. Unless the experience of reward and beauty are explored in the same subjects and experiments and a conjunction analysis used, it is difficult to determine with precision whether the identical parts of mOFC are involved in these different experiences. As well, if identical parts are involved, it is difficult to determine from imaging experiments whether the same groups of cells are recruited during these different experiences, which nevertheless share certain features – being rewarding, pleasant or beautiful – in common. Meta-analyses such as the ones provided by Peters and Büchel (2010) or Kühn and Gallinat (2012), though very useful, are not much help in this context; such meta-analyses help locate the general brain regions active with specific experiences but are not presently capable of pinpointing whether the identical regions are active.

## AESTHETIC EXPERIENCE AND JUDGMENT

mOFC is as well active during aesthetic judgments (Ishizu and Zeki, 2013), which is implied in the experience of beauty. Whether there are separate sub-divisions within mOFC that mediate, for example, the judgment of beauty as opposed to its experience, remains to be seen; indeed whether the judgment of beauty comes before its experience or whether the two occur simultaneously, both spatially and temporally, remains an issue to be addressed. Equally, whether one can distinguish neurobiologically between the pleasure derived, for example, from listening to a light operetta and the more demanding but ultimately more rewarding symphonic works of Beethoven is not currently known and nor has the question been addressed experimentally. For the moment, it is sufficient for my argument here to highlight the fact that the mOFC is always active when humans experience beauty. Such a definition does not, of course, define beauty and nor is it the aim of neuroesthetics to do so, its more limited aim being to understand the neural mechanisms that allow us to experience beauty.

## QUANTIFYING THE SUBJECTIVE EXPERIENCE OF BEAUTY

**FIGURE 2:**
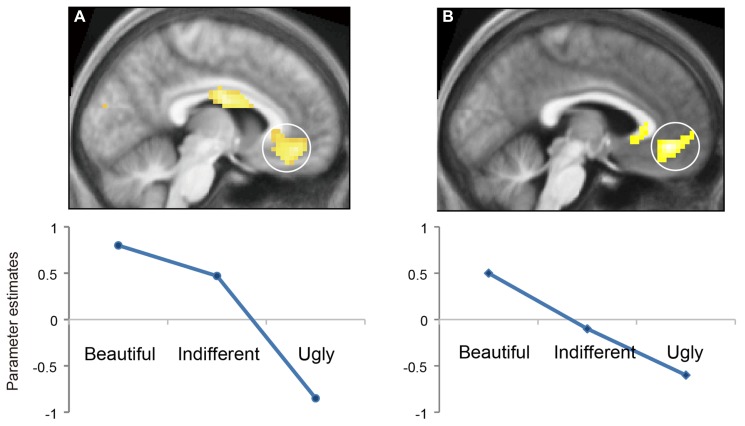
**Modulation of cortical activity by aesthetic rating.** Averaged parameter estimates showing modulation by beauty rating (beautiful, indifferent, and ugly) in mOFC for **(A)** visual stimuli and **(B)** musical stimuli. A linear relationship with beauty rating was observed in both conditions. From Ishizu and Zeki (2011).

The strength of activity in the mOFC is proportional to the declared intensity of the experience, the more intense the declared experience, the more intense the activity (**Figure 2**). The graphs of **Figure 2** are derived from the observed intensity of activity in the mOFC against the declared intensity of the experience of beauty, averaged across subjects, without reference to the actual paintings or musical excerpts that gave rise to the experience. Similar parametric relationships have been observed between strength of activity in mOFC and experience of reward and pleasure in the articles cited above. This is interesting for two reasons. In the first place it gives a neurobiological answer to a question which, though not raised by Bell, has nevertheless been regarded as central in the philosophy of aesthetics, namely whether there can be objective judgments of aesthetic value (Graham, 2000). The novelty here is that the objective judgment relates directly to strength of activity in a precise locus in the brain. It may thus be called subjective, to the extent that it is activity in individual brains relating to private (if declared) experiences, although what one person experiences as beautiful is not necessarily the same as what another person experiences. But it is also objective to the extent (a) that whenever a subject experiences beauty, regardless of source and of culture and education, the mOFC is active and (b) that the activity there is detectable and quantifiable. How this strength of activity is related to strength of activity in what we may loosely call the “sensory” areas of the brain remains to be determined (see below).

It is trite to state that art and beauty are not the same, nor is it the function of art to be solely concerned with beauty, a separation forcefully emphasized by Marcel Duchamp when he sent a urinal to an art exhibition. Hence these experiments are concerned with the experience of beauty alone, not with art. Many of the pictures viewed and the musical excerpts listened to by subjects would be considered great works of art by experts, but they were not necessarily experienced as beautiful by subjects. This raises another interesting point, which future experiments need to address. Bell saw beauty as a confusing term, which he wanted to avoid. He wrote, “But most of us, however strict we may be, are apt to apply the epithet “beautiful” to objects that do not provoke that peculiar emotion produced by works of art. Everyone, I suspect, has called a butterfly or a flower beautiful. Does anyone feel the same kind of emotion for a butterfly or a flower that he feels for a cathedral or a picture; surely, it is not what I call an aesthetic emotion that most of us feel, generally, for natural beauty…some people may, occasionally, see in nature what we see in art, and feel for her an aesthetic emotion; but… as a rule, most people feel a very different kind of emotion for birds and flowers and the wings of butterflies from that which they feel for pictures, pots, temples, and statues” (my ellipsis).

## DETACHMENT OF THE AESTHETIC EMOTION FROM CULTURE AND COGNITION

Bell’s aesthetic formulation depends upon objects with the power of arousing the “aesthetic emotion” having a *Significant Form*, which he defines as a particular arrangement of lines and colors, without specifying it further, for “it need be agreed only that forms arranged and combined according to certain *unknown and mysterious* laws do move us in a particular way, and that it is the business of an artist so to combine and arrange them that they shall move us” (my emphasis). He was not alone in thus emphasizing line and color. Piet Mondrian also emphasized that he wanted to create beauty through line and color (alone). In a letter, he wrote, “I construct line and color combinations, in order to express general beauty with the utmost awareness…. I believe it is possible that, through horizontal and vertical lines constructed with awareness, but not with calculation, led by high intuition, and brought to harmony and rhythm, these basic forms of beauty…can become a work of art” (Mondrian, letter to H. P. Bremmer, 1914). One of the neurobiologically powerful arguments in Bell’s thesis is that those *unknown and mysterious laws* have to be something fairly basic and unrelated to learning, memory, and cultural background, an interesting counterpoint to the many who insist that the experience of beauty is mandatorily tied to culture and education. He writes, “Imperfect lovers [of art] bring to art and take away the ideas and emotions of their own age and civilization. In twelfth century Europe a man might have been greatly moved by a Romanesque church and found nothing in a T’ang picture. To a man of a later age, Greek sculpture meant much and Mexican nothing, for only to the former could he bring a crowd of associated ideas to be the objects of familiar emotions. But the perfect lover [of art], he who can feel the profound significance of form, is raised above the accidents of time and place. To him the problems of archeology, history, and hagiography are impertinent. If the forms of a work are significant its provenance is irrelevant. Before the grandeur of those Sumerian figures in the Louvre he is carried on the same flood of emotion to the same aesthetic ecstasy as, more than 4000 years ago, the Chaldean lover was carried. It is the mark of great art that its appeal is universal and eternal.” Again, without saying so explicitly, he is implying that its appeal is “universal and eternal” because something in the (mental and neural) biological constitution of all humans makes us receptive to it.

Implicit in the above quote and in the list that Bell gives is the assumption of what Immanuel Kant (Kant, 1790/1987) called the *sensus communis* which unites artist and viewer irrespective of culture and experience, since the objects Bell enumerates come from different times and cultures and evoke a general emotion that is common “to all ages, and peculiar to none.” Both in *Art* and in his later book *Since Cézanne* (Bell, 1922), he is at pains to emphasize that he is not linking it to intellect and learning, but to something more basic, something “primitive” that is common to all humankind. To him, intellect is the enemy of the aesthetic emotion and “the last to feel aesthetic emotion is the historian of art” since “The habit…of seeing intellectually instead of seeing emotionally, accounts for the amazing blindness, or rather visual shallowness, of most civilized adults.” When we view works of art, “it is with their aesthetic and not with their cognitive value” that we should be concerned, thus dissociating the aesthetic from the cognitive, including cultural, element. An artisan can, he believes, learn something from masterpieces “provided there is no cultivated person at hand to tell him what to feel, *or to prevent him feeling anything by telling him to think*” (my italics). He writes, “Only artists and educated people of extraordinary sensibility and some savages and children feel the significance of form so acutely that they know not how things look. These see, because they see emotionally.” To appreciate “a work of art we need to bring with us nothing from life, no knowledge of its ideas and affairs, no familiarity with its emotions.” Nor is the artist, the creator, exempt from this, to the extent that any theoretical, scientific or literary associations are, according to Bell, the surest route to compromise artistic creativity and imperil the “aesthetic emotion” that their creations might otherwise arouse. This is something, he believes, that primitives understood since they “neither create illusions, nor make display of extravagant accomplishments, but concentrate their energies on the one thing needful – the creation of form. They thus have created the finest works of art that we possess”; “*Il nous faut les barbares*” [we need the barbarians] he therefore quotes André Gide approvingly as saying (Bell, 1922). A characteristic of “primitive” art is that it does not preoccupy itself with “exact representation and ostentatious cunning” since even though the “representative element in a work of art may or may not be harmful, always it is irrelevant.” And all those who practice art with some kind of intellectual overtone or undertone are doomed: Per Krogh “started work under three crippling disabilities – a literary imagination, natural facility, and inherited science”; Van Gogh was a preacher and therefore “too often his delicious and sensitive works of art are smeared over, to their detriment, with tendentious propaganda”; Vlaminck “had the misfortune to learn a recipe for making attractive and sparkling pictures; he is now, I understand, in retirement trying to unlearn it,” and so the list goes on. What matters, therefore, is the skeleton, not the embellishments, “that which is left when we have stripped a thing of all its associations, of all its significance as a means.”

This emphasis on something detached from intellect and cognition, something more primitive and universal, can be found in others since Bell. In literary art, Marcel Proust (1971) began his posthumously published book, *Contre Sainte-Beuve*, by stating, “*Chaque jour, j’attache moins de prix à l’intelligence. Chaque jour je me rends mieux compte que ce n’est qu’en dehors d’elle que l’écrivain peut ressaisir quelque chose de nos impressions passées, c’est-à-dire atteindre quelque chose de lui même et la seul matière de l’art”^1^, giving to involuntary memories a primary place and to the intellect a secondary one in artistic creativity, a view he developed in his masterpiece. This insistence on the primacy of instinct “…touche à de très importants problèmes intellectuels, peut-être au plus grand de tous pour un artiste, à cette infériorité de l’intelligence…Et cette infériorité de l’intelligence, c’est tout de même à l’intelligence qu’il faut demander de l’établir. Car si l’intelligence ne mérite pas la couronne suprême, c’est elle seule qui est capable de la decérner. Et si elle n’a dans l’hiérarchie des vertus que la seconde place, il n’y a qu’elle qui soit capable de proclamer que l’instinct doit occupier la première”^2^*. Proust was thus emphasizing the primacy of instinct and intuition in the creation of a work of art but the primacy of intellect in discussing it.

Francis Bacon always emphasized in his work that he wanted to “assault the nervous system” with the “rawness of the image,” to deliver a “visual shock” and that his work was devoid of narrative, insisting that he had “no story to tell” (Zeki and Ishizu, 2013). He wanted to produce an immediate emotional impact on the nervous, “before things got spelled out in the brain” (Peppiatt, 1996). Hence he, too, was giving the intellect and cognition a secondary place in artistic appreciation. More recently, Freedberg and Gallese (2007) have challenged the primacy of cognition in responses to art and have related such responses to phylogenetically acquired embodied simulations, that is to a correspondence between the viewed work and the (universal) brain templates that they activate, emphasizing in particular the actions represented and their brain templates within the context of mirror neuron systems. This is a perfect recipe for neuroesthetic investigation, which also is concerned more with neural essentials, while not denying that culture is an important component in the experience of beauty. It is when he writes of Cézanne that Bell (1922) comes close to admitting, perhaps without realizing it, that these mysterious laws refer to something in our own mental or neural constitution: “Cézanne was direct because he set himself a task which admitted of no *adscititious* flourishes – the creation of form which should be entirely self-supporting and intrinsically significant…To achieve it he was prepared to play the oddest tricks with natural forms – to distort” for “What is important in his art is, of course, the *beauty of his conceptions*… indifference to verisimilitude is but the outward and visible sign of this inward and spiritual grace” (my ellipsis and emphases). He is therefore referring unwittingly to concepts of form in the brain, to which external forms became subservient in Cézanne’s work. Reality can therefore be distorted through the conceptions of an artist but these distortions are only distortions of the external reality; they maintain the significant form because significant form is a part of our mental, and therefore neurobiological, constitution. This, in a sense, unknowingly anticipates a quest of neuroesthetics, which is to learn about those “mysterious laws” which are at the base of the experience of beauty regardless of culture and upbringing and are, therefore, to a large extent biologically determined.

It is therefore interesting to emphasize that, in our experiments referred to above, the objective identification of the brain locus, whose intensity of activity correlates with the declared intensity of the experience of beauty, is indifferent to culture, upbringing, education, national or ethnic values. The subjects participating in these experiments came from a variety of ethnic and cultural backgrounds. Not all paintings or musical excerpts were uniformly experienced as beautiful by all subjects but, whenever a subject experienced beauty, there was, as a correlate, activity in the mOFC. Without denying the importance of culture and education in shaping the experience of beauty, the importance of this neurobiological definition transcends cultures and is universal. It is, in Bell’s terms, a capacity to experience beauty that applies “to all ages, and [is] peculiar to none.” Hence we may speak of Immanuel Kant’s *sensus communis* in a somewhat different sense than intended by him – as a common ability to experience beauty which can be objectively ascertained as a common correlate of activity in a specific area of the brain in all humans.

## “SIGNIFICANT FORM” AND THE AESTHETIC EMOTION

The extent to which Bell was a formalist, looking for qualities in the objects themselves, even in spite of his subjective approach, becomes clear when he writes of his list, given above, that, “In each, lines and colours combined in a particular way, certain forms and relations of forms, stir our aesthetic emotions. These relations and combinations of lines and colours, these aesthetically moving forms, I call ‘Significant Form’, which is ‘the one quality common to all works of art’.” He does not specify what “Significant Form” means in terms of mathematics or music but his statement, that at times he can appreciate music as “pure musical form,” as “sounds combined according to the laws of a mysterious necessity,” implies that he had in mind an unspecified formal objective structure to music which also arouses the “aesthetic emotion.”

“Significant form” is therefore a primordial quality and, although he never says so explicitly, it becomes clear that it is also a property of the work itself. This leads to some confusion. For, to arouse an emotion, lines, forms, and colors must be perceived first, a function of the perceptive system of the brain. Hence, interposed between lines, forms, and colors and the emotion that they arouse is perception. I agree with Gould (Gould, 1994) that what Bell calls the aesthetic emotion is better described as the aesthetic perception; his entire argument acquires much greater force and coherence, as well as neurobiological relevance, when viewed in that light. Neurobiology, in turn, is better guided to ask how visual perception can arouse the aesthetic emotion. Although Bell does not allude to the definition of beauty given by Edmund Burke centuries earlier, there is in that definition as well something (perception) that is interposed between the objects of beauty and the emotion that they arouse: “Beauty is, for the greater part, some quality in bodies acting mechanically upon the human mind by *the intervention of the senses*” (Burke, 1757; my italics).

This raises a neurobiological challenge which can be summed up as follows: are there any arrangements of lines, forms and colors (or indeed of other visual attributes) and the relations between them which would adhere to neural laws of “mysterious necessity” and thus satisfy the “unknown and mysterious laws” of our perceptive system sufficiently to arouse the aesthetic emotion? More broadly, do such arrangements result in a pattern of activation in the perceptive areas that can, by some criterion or another, be said to correspond to an “aesthetic perception” and what is the relation, in neural terms, between an aesthetic perception and the “aesthetic emotion” that it arouses?

## OBJECTIVE AND SUBJECTIVE

Neurobiologically, the issue revolves around what these “mysterious laws” can be and how the artist succeeds in tapping them. One of the weaknesses in Bell’s formulation is, as Gould has so well put it, the failure to recognize that “the perception of significant form is one thing, the accompanying pleasure another.” And the task for neurobiology is to try to establish what combinations of lines and colors, and of other visual attributes, activate the perceptual areas in a way that they arouse pleasure and, as a correlate, activity in the mOFC, since it is evident that not all combinations of lines and colors arouse the aesthetic emotion or lead to activity in the mOFC. Since Bell’s arena was mainly the visual, it is perhaps useful to begin by asking whether there is, or there can be, any common quality in all visual objects that provoke the “aesthetic emotion.” The question can be more precisely phrased thus: what is the pattern of activation in visual areas that would recruit activity in the pleasure and reward centers of the brain, and particularly the mOFC, which correlates with the aesthetic emotion?

## WHY THERE CANNOT BE SINGLE CHARACTERISTIC(S) THAT AROUSE THE AESTHETIC FEELING

It is common knowledge that many, including Vitruvius, Alberti, and Leonardo, have sought a characteristic or characteristics of objects that render them beautiful, but without any firm conclusion. This is partly due to the fact that characteristics such as proportion and symmetry, though applicable to architecture, cannot be applied to attributes such as color and it is significant that Bell does not mention them either. It is also partly due to strong cultural influences and predilections that often emphasize different, and contradictory, characteristics with which Bell, incidentally, is specifically not concerned since his concern is with the “universal and eternal.” While symmetry and regularity have been, at one time or another, thought to be important in Western culture, the Japanese aesthetic places a high premium on asymmetry and irregularity (*fukinsei*). And while the unfinished and the unstated are greatly valued in Chinese and Japanese cultures, as provoking (melancholy) beauty, the unfinished has received, at different times, a very mixed reception in Western art, as criticisms of, for example, Cézanne’s work because of its “unfinished” status demonstrate (Zeki, 2008). It is, therefore, idle to suppose that one can identify a single characteristic or a single set of characteristics that renders objects as diverse as the list that Bell gives beautiful.

But there is perhaps another, neurobiological, factor why there is no single characteristic (or characteristics) that turn works of visual art into something beautiful enough to provoke the “aesthetic emotion.” That factor lies in the functional organization of the visual brain.

Let us for a moment assume that when Bell writes of lines, forms, and colors, and certain combinations of the three, he literally means lines, forms, and colors, but ones that are assembled in certain emotionally (or, better still, perceptually) provocative ways. In his artistic exploration of form, Mondrian (and others) supposed that the oriented line, and especially the vertical and horizontal, is the essential constituents of all forms and that form can be defined as “the plurality of straight lines in rectangular opposition” (Mondrian et al., 1986). A neurobiological problem intrudes here, for the relationship of lines to more complex forms is enigmatic; ever since the discovery of orientation selective cells (cells that respond to lines of specific orientation) in the visual brain (Hubel and Wiesel, 1959), neurobiologists have supposed (Riesenhuber and Poggio, 1999, *inter alia*), though without much supporting experimental evidence, that such cells are the physiological building blocks of forms in the brain, in hierarchical fashion, with groups of cells in each visual area analyzing the same information as temporally antecedent groups of cells in the same visual area but at a higher level of complexity, or with cells in the different visual areas that constitute a single hierarchical “form processing” chain analyzing the same information at a more complex level than the cells in the antecedent area (Hubel and Wiesel, 1977). There is no doubt of the existence of a hierarchical processing within visual areas (Alonso and Martinez, 1998; Martinez and Alonso, 2001). But, even today, it is not at all certain that, if orientation selective cells are the precursors for the elaboration of complex forms in the brain, how that elaboration occurs, in other words how orientation selective cells are used to construct more complex objects, such as the ones that area lateral occipital cortex (LOC) responds to (Grill-Spector et al., 2001; see above). Hence the relationship of line to form is not at all clear. Lines may be arranged in a pleasing way but when combined to produce more complex forms, the result may not be so pleasing. Nor is the relationship of line to form the only enigmatic one in neurobiological terms; so is the relationship of form to color, the relationship of moving to stationary stimuli, and of both to color.

It is obvious that any picture, even one combining line and color alone, as perhaps in the work of Piet Mondrian or Yves Klein, to mention just two, may have a certain combination and configuration of lines which may activate the areas containing heavy concentrations of orientation (line) selective cells optimally or specifically, and a certain combination of colors that may activate the cerebral areas specialized for the perception of colors sub-optimally, or vice versa. In fact, the examples that Bell enumerates in his list above are much more complex – line and color are only two among many other components. The masterpieces of Cézanne and Piero della Francesca are representational and contain faces, bodies, landscapes, and simulated depth on a flat two dimensional canvas – features that are known to be processed in separate visual areas. Here again, one may find configurations that are optimal or specific for stimulating one of the specialized visual areas but not the others.

## “SIGNIFICANT CONFIGURATION” IN DIFFERENT VISUAL DOMAINS

**FIGURE 3:**
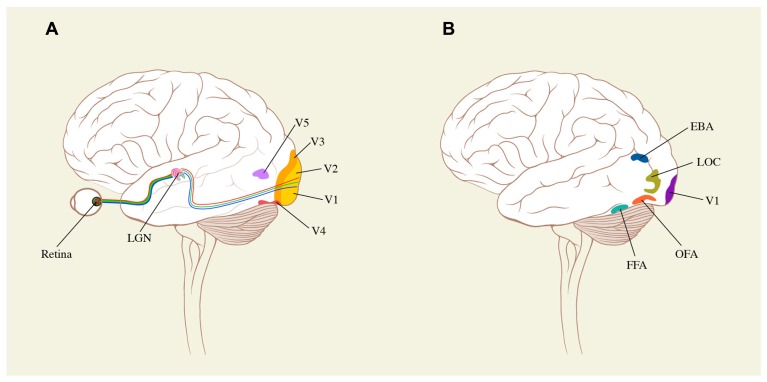
**Schematic surface drawings of the brain to indicate (A) the relative positions of the early visual areas (V1–V5) referred to in the text and (B) the positions of areas critical for face (OFA and FFA), body (EBA) and object (LOC) representation**. For further details, see text.

In terms of modern neurobiology, then, another weakness of Bell’s theory is that, even when writing of the visual domain alone, his significant form is constituted by, and restricted to, lines, forms, and colors and their arrangement in relation to one another. For although there is no disputing their importance, these attributes are not only processed by separate neural systems in the brain (Zeki, 1978a, 1978b; DeYoe and Van Essen, 1985; Shipp and Zeki, 1985; Livingstone and Hubel, 1988; Zeki et al., 1991) but also are not the only attributes which can arouse an “aesthetic emotion.” A much more complex picture of the visual brain, and how it functions, has emerged over the past 50 years (see **Figure 3**). The primary visual cortex, or visual area V1, is the principal but not sole cortical recipient of visual signals from the retina. Surrounding it are several visual areas which receive specialized visual signals from it and from other visual centers, both cortical and sub-cortical. Apart from one area (V2) which, like V1, appears to have all the primary visual attributes represented in it (Zeki, 1978a; DeYoe and Van Essen, 1985; Shipp and Zeki, 1985; Livingstone and Hubel, 1988) the other areas surrounding V1–V2 are specialized to process different attributes of the visual world. Among these specializations are ones for visual motion (based on area V5 and its satellites – the V5 complex; Zeki, 1974; Watson et al., 1993; Orban et al., 1995), the V4 complex, critical for the perception of colors (Zeki, 1973; McKeefry and Zeki, 1997; Bartels and Zeki, 2000; Wade et al., 2002; Goddard et al., 2011) and form-in-association with color (Zeki, 1993); and the V3 complex (comprising V3, V3A, and V3B; Smith et al., 1998; Press et al., 2001), specialized for the perception of forms, especially dynamic ones (Zeki et al., 1991, 2003; Zeki, 1993). Other areas are specialized for the perception of faces (Sergent et al., 1992; Kanwisher et al., 1997) and facial expressions (Yang et al., 2002; Vuilleumier and Pourtois, 2007; Derntl et al., 2009) as well as human bodies (Downing et al., 2001), although there is some disagreement about whether faces are processed by separate areas or by separate groupings within a larger area that also processes objects (Haxby et al., 2001). Evidence suggests that there are other cerebral visual areas which are specialized for other attributes. One of these is the LOC (Malach et al., 1995), located more anteriorly in the visual brain, and critically involved in object recognition, although how its properties are elaborated from the orientation selective cells of V1 and V2, a common if unproven supposition (see above), remains unknown. The total number of visual areas in the brain has not yet been determined, and more areas continue to be discovered; previously established areas are sometimes subdivided into further areas. But the multiplicity of visual areas in the brain is now established beyond doubt (**Figure 3**).

The functions that I have attributed to these areas are not necessarily their only functions. It is certain that there are common functions that they all share. Among these is attention, and all areas so far studied have been found to have attentional mechanisms built into them (Boynton, 2009; Jehee et al., 2011). It is probable that each area has additional functions. What is reasonably well established is that damage to the brain, when restricted to one of these areas, results in an imperception for the attribute for which that area is specialized. Damage to the V4 complex leads to the syndrome of cerebral achromatopsia (Meadows, 1974a; Mollon et al., 1980; Zeki, 1990) when the world is no longer perceived in color but in “dirty” shades of gray; damage to the V5 complex results in the syndrome of cerebral akinetopsia (Zeki, 1991), leading to an incapacity to see objects in the visual world when they are in motion; damage to the area specialized for face perception leads to the syndrome of prosopagnosia, an incapacity to recognize people by their faces (Pallis, 1955; Meadows, 1974b; Steeves et al., 2006). In fact, even the form system of the visual brain, widely regarded as a single hierarchical system (Felleman and Van Essen, 1991) that elaborates complex forms from more simple ones, and especially from cells that respond to lines of specific orientation, may in fact be composed of several parallel systems. The evidence for this comes from two sources – brain imaging experiments and clinical observations. The former shows that increasingly more complex forms constituted from lines (lines, angles, and rhomboids) do not activate visual areas with heavy concentrations of orientation selective cells more strongly than the simple lines from which they are constituted, as would be expected if complex forms are built up serially from simpler ones; instead all three forms activate all such areas with equal strength. Moreover, the latency of activation of these areas with lines and rhomboids is also much the same (Shigihara and Zeki, 2013), rather than what might be expected from the hierarchical doctrine of form construction, that more complex forms should activate the visual areas with longer latencies than simpler forms. This suggests that the perceptual hierarchy of forms is not mirrored by a spatial or temporal hierarchy of form processing in the brain. In Gestalt language, “the whole is other than the sum of the parts.”

The clinical evidence shows that an agnosia for complex shapes and objects need not be accompanied by an agnosia for simple line representation of the same shapes (Humphreys and Riddoch, 1987) and, conversely, that agnosia for simple line drawings of complex shapes need not be accompanied by an agnosia for the complex shapes themselves (Hiraoka et al., 2009). These are some of the best documented examples of specific visual syndromes resulting from damage to specific, specialized, visual areas, although there are other ones. Collectively, this evidence supplements the anatomical and physiological evidence in favor of a functional specialization in the visual brain.

Hence, Bell’s formulation, though restricted to lines and colors, forces us to go beyond and, in light of modern neurobiology, leads us to the broader question of whether there is a significant configuration for other visual attributes, such as faces, bodies and objects – attributes that are processed in distinct visual areas – that arouse the aesthetic emotion and, if there are, where in the brain these configurations are detected, through what neural means, and what the relationship is between the hypothetical neural activity in these specialized areas and activity in the mOFC which is a correlate of the experience of beauty.

## SEPARATE NEURAL SYSTEMS FOR GROUPING VISUAL SIGNALS ACCORDING TO ATTRIBUTE

**FIGURE 4:**
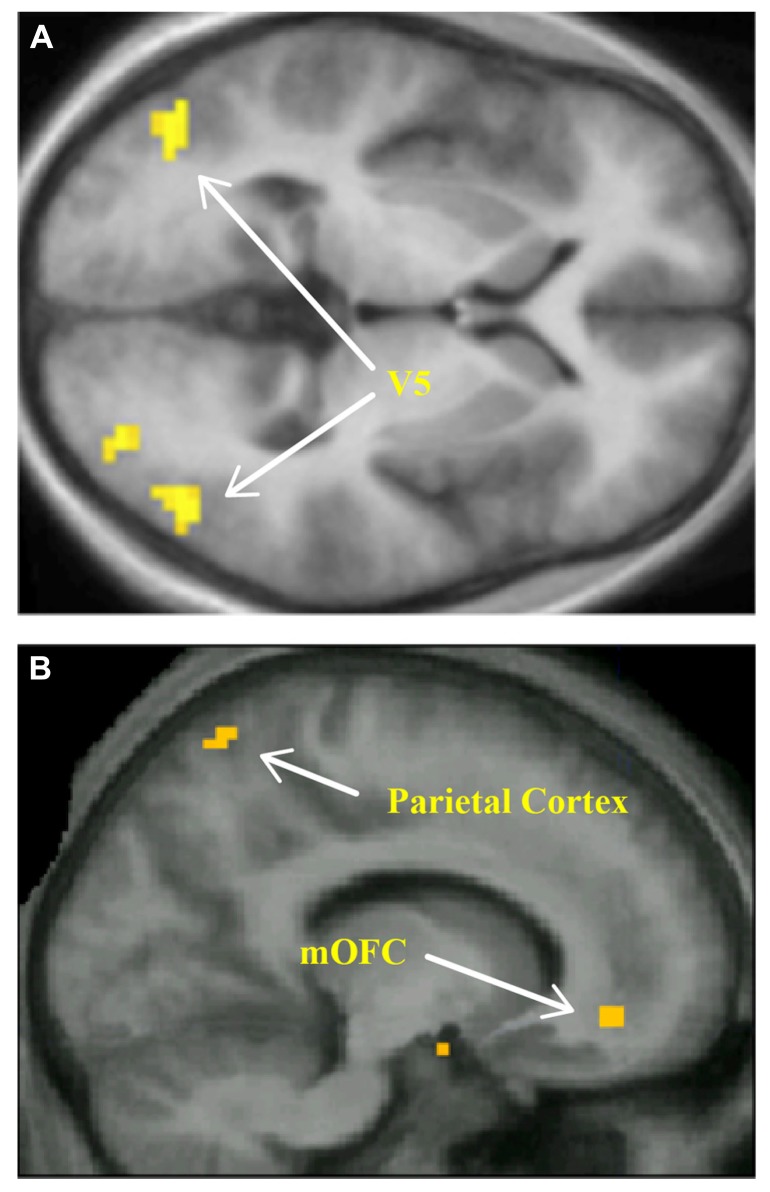
**Among the brain areas where the intensity of activity is proportional to the preferred pattern of kinetic stimuli are areas V5, seen here in a horizontal brain section (A)** and the parietal cortex, shown in a sagittal section **(B)**. The activity in V5 that correlated with the preferred ratings of these kinetic patterns also correlated with activity in the mOFC **(B)**. From Zeki and Stutters (2012).

Bell wrote of the “combination” of lines and colors and it is interesting to note that grouping of stimuli (which is only loosely equivalent to the combination of lines and of colors that Bell speaks of) is also processed separately in the brain. Grouping (or segmentation) is a critical process in visual recognition (Wertheimer et al., 1950/1923; Palmer, 2002). Anatomical and imaging experiments show that the processes underlying grouping for different attributes are separated within the parietal cortex. One part of the parietal cortex seems to be involved in grouping of stimuli according to color and a separate, more lateral though adjacent, part involved when they are grouped according to direction of motion (Zeki and Stutters, 2013), reflecting the anatomical arrangement of connections from V4 (color) and V5 (motion) to the parietal cortex, the input from V4 lying medially to that from V5 within parietal cortex (Shipp and Zeki, 1995; **Figure 4**). It seems likely that a separate but contiguous part of the parietal cortex is involved in grouping of visual signals according to form (Braddick et al., 2000). Moreover, these same but separate regions of parietal cortex appear to be involved in forming visual concepts based on color and motion (Cheadle and Zeki, 2013, submitted). Bell’s formulation thus raises the interesting, and so far un-addressed, issue of whether there is a quantitative relationship between the intensity of activity in these parietal cortex sub-divisions and aesthetically satisfying groupings of color, motion, and other visual attributes that are separately processed and whether there is any relationship between the activity produced by aesthetic groupings and activity in the mOFC.

## THE PROJECTION OF FUNCTIONAL SPECIALIZATION IN TIME

A specialization for processing different visual attributes can also be surmised from psychophysical evidence, which shows that we become conscious of the color of a visual stimulus before we become conscious of its orientation or of its direction of motion (Moutoussis and Zeki, 1997; Viviani and Aymoz, 2001; Arnold and Clifford, 2002; Linares and Lopez-Moliner, 2006). The temporal difference is not insignificant – color is perceived before motion by about 80 ms, an inordinately long time in neural terms. Hence the observed functional specialization is projected in time, and leads to a perceptual asynchrony in vision. The consequences of this asynchrony are important for understanding how the visual brain functions and may also have some relevance in the context of Bell’s formulations. Since attributes such as color, form and motion are processed by distinct and geographically separate areas of the visual brain, it follows that visual consciousness is distributed in space. Since we become conscious of different attributes at different times, it follows that visual consciousness is also distributed in time. From which it follows that there are several visual consciousnesses which are distributed in time and space (Zeki, 2003). Hence, in temporal terms, these different attributes of vision are also handled separately in the visual brain, raising neurobiological questions in terms of Bell’s formulation. Is a grouping according to color also made faster than a grouping according to, say, motion and, if so, would an aesthetically satisfying grouping according to color be perceived before an aesthetically satisfying grouping according to motion or some other attribute? It is plausible, and interesting, to suppose that combinations that satisfy some more primitive significant configuration, and are found to be more aesthetically pleasing, may be processed more rapidly than those which, not coming as close to satisfying a significant configuration, are found to be less satisfying aesthetically.

But Bell of course goes beyond lines and colors, to their combination. How lines and colors are combined in the brain to give us our “unitary” experience of a composition, and thus arouse the “aesthetic emotion,” remains a mystery. Indeed, it is not even clear that they are combined or that our experience is unitary. Nor is it clear how other attributes are combined to give us our unitary experience. In a composite picture, it is almost impossible to concentrate on the color and the shape simultaneously and psychophysical evidence (see above) that, over brief windows of time, separate attributes are processed and perceived separately and are thus misbound (Moutoussis and Zeki, 1997), suggests that they may not be combined, at least over such brief windows of time. Yet there is no denying that the combination of the two, as well as of other attributes, can be critical to the aesthetic value of a painting nor is there any denying that lines and colors, as well as other attributes, are combined over the longer term. It is a puzzle that neurobiology has not yet solved, but one possibility that must be entertained is that such binding does not occur by physiological interaction between separate perceptual areas but is post-perceptual and may involve memory. This is interesting from another point of view. The aesthetic emotion that an object arouses does so when viewed holistically, but it is not at all certain that its different components are all perceived at the same time. A portrait painting may have an aesthetically appealing face and a colored background; while there is little doubt that the face itself will be perceived holistically (see below), it is likely (given the evidence above) that the colors of the background will be perceived on a different time-scale, thus raising the question of how all the elements are ultimately bound together, emphasizing once again that the whole is other than the sum of the parts.

## SIGNIFICANT CONFIGURATION AS A BETTER TERM

If we follow Gould in replacing “aesthetic emotion” with “aesthetic perception,” we might do well to ask speculatively whether the mysterious laws that Bell speaks of consist of preferred activation patterns in the relevant visual areas, the result of certain combinations of the relevant attribute which are biologically determined to be more significant and hence independent of culture and learning. The mysterious laws that the artist taps becomes, then, his or her capacity to create forms that activate the relevant visual areas either optimally or specifically, by which I mean activate them in a way that is different from that obtained by stimuli that lack the significant configuration. Perhaps only when so activated are the “sensory” areas of the brain able to arouse the aesthetic emotion. These hypothetical conjectures may not in fact be so improbable, as I discuss below. Here, I would like to propose that the term “*significant configuration*” may be better tailored to what we know about the visual brain than Bell’s “significant form,” since the latter, by his definition, is restricted to lines and colors and their combinations. The term significant configuration is not so limited; it could apply to any attribute – for example, faces, bodies, or kinetic stimuli. All one has to do is to determine whether there is a significant configuration in each of these different domains that activates the area maximally, or optimally, or in a way that might be referred to as aesthetically. When considering aesthetic perception, however, we have to consider both positive and negative effects since, to experience something as beautiful, implies not experiencing it as ugly or neutral, and vice versa. Overall, experiencing something as beautiful leads, *inter alia*, to activation of the mOFC while experiencing something as ugly or threatening leads to activation of other structures, principally the amygdala (Morris et al., 1996) and insula (Krolak-Salmon et al., 2003). But objects, and in particular faces and bodies, are perceived holistically (Tanaka and Farah, 1993; Aviezer et al., 2012) and hence it is a significant configuration of the various constituents, the “combinations” that Bell speaks of, that qualifies a face as happy or ugly, a body as joyful or threatening, and a kinetic stimulus as pleasing or neutral.

## A SIGNIFICANT CONFIGURATION FOR THE VISUAL MOTION SYSTEM

It is perhaps instructive to begin by looking at the simplest of stimuli, that of visual motion, and asking whether there is a significant configuration in a pattern of kinetic stimuli that is preferred by subjects and that privileges it over other kinetic patterns. By privileging it, I mean (a) that it leads to a different pattern of activation than other kinetic stimuli and (b) that it leads to a correlated activity in the mOFC. A different pattern of activation can be one of three possibilities: (*a*) a more intense response from the visual areas specialized in the processing of visual motion; (*b*) a specific pattern of activation that engages a different group of cells than the ones engaged by other patterns (*a* and *b* are not mutually exclusive); (*c*) an optimal response, which is not the same as a more intense response.

Area V5, one of the most intensively studied areas of the brain, is specialized for visual motion; its cells respond to motion, most of them being directionally selective (Zeki, 1974; Van Essen et al., 1981; McKeefry et al., 1997; Bair and Movshon, 2004; i.e., responsive to one direction of motion and not to motion in the opposite direction). Other areas in the brain that respond robustly to motion are those that constitute the V3 complex (V3, V3A/V3B), which may be involved in the perception of dynamic forms (Zeki, 1993; Zeki et al., 2003). Collectively, these areas provide fertile ground for testing the supposition that subjective preference for a given category of visual stimuli – in this case kinetic stimuli – results in a different pattern of activation than non-preferred kinetic stimuli (Zeki and Stutters, 2012).

**FIGURE 5:**
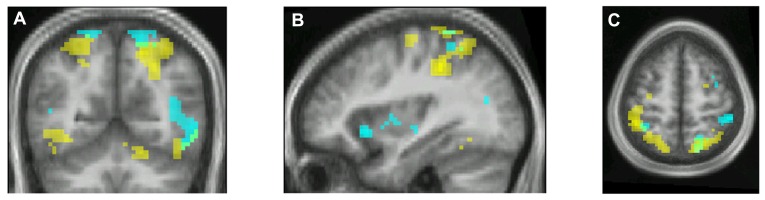
**Overlaid contrasts showing conjunction of activity produced by the contrasts color grouping > no grouping (yellow) and motion grouping > no grouping (cyan) as seen in coronal (A), parasagittal (B) and horizontal (C) sections through the brain**. From Zeki and Stutters (2013).

Recent experiments demonstrate that there are certain combinations of dots in motion (kinetic stimuli) that are preferred by humans over other combinations, and that the preferred combinations activate the areas specialized for visual motion, among them the V5 complex, as well as other areas which may be involved in the perception of dynamic forms, more powerfully than the non-preferred combinations (Zeki and Stutters, 2012; **Figure 5**). The strength of activity in these areas, as well as in parietal cortex, is proportional to the intensity of the declared subjective preference for the patterns of kinetic dots. Hence there is a direct and quantifiable relationship between declared preference and strength of activation in the relevant visual area. Of the three possibilities given above, it follows that the observed difference here is a stronger activity (which can be objectively verified) with a significant configuration of the stimuli (which can also be objectively specified). Moreover, viewing the preferred kinetic stimuli also leads to activity in the mOFC (Zeki and Stutters, 2012). It is as if a significant configuration of kinetic elements activates the relevant visual areas (V5 and V3A) optimally or selectively (aesthetic perception) which also correlates with activation of the mOFC (aesthetic emotion). I emphasize that there are other stimuli – in particular chaotic motion – which give very strong activation of V5 (ffytche et al., 1995). Hence when I speak of stronger activation above, I only mean stronger activation than non-preferred kinetic stimuli. This raises the possibility that it is not the strongest or maximal activity that correlates with preference but rather a specific activity that becomes optimal when stimuli of the right significant configuration are viewed. How this stronger optimal activation is translated into an aesthetic appreciation is not certain; aesthetic preference correlates with activity in other parts of the brain (see below). But this demonstration nevertheless opens up the possibility that each of the specialized visual areas may have a certain, primitive, biologically derived combination (by which, following Bell, I mean one that is not subject to cognition, cultural influences, and learning) of elements for the attribute that it is specialized in processing, and that the aesthetic perception (which ultimately leads to the aesthetic emotion) is aroused when, in a composite picture, each of the specialized areas is activated preferentially.

The above is perhaps the simplest example of a significant configuration that not only leads to stronger or privileged activation of the early perceptual areas (aesthetic perception) but also has, as a correlate, activity in the mOFC (aesthetic emotion). In fact, other experiments have shown that there is an increase of activity in the occipital cortex as a whole, the fusiform gyrus (comprising the visual areas) and the caudate nucleus when subjects view abstract and representational paintings that they prefer (Vartanian and Goel, 2004). Hence, it is possible to speculate that “aesthetic perception” (to use Gould’s term) has, as a correlate, an enhanced and possibly specific activity within early visual perceptive areas. What precise neural conditions recruit activity in the mOFC remains to be seen. The determination of these conditions may link aesthetic perception to aesthetic emotion.

## SIGNIFICANT CONFIGURATIONS FOR FACES AND BODIES

Outside motion, it is clear that there are essential configurations (to be distinguished from significant configurations) which are critical for activating the relevant visual areas or systems. Among the most obvious are faces and bodies. There is no doubt that there is an essential configuration that qualifies a set of stimuli as constituting a face. New-born infants apparently have an innate predisposition for recognizing faces even a few hours after birth (Goren et al., 1975; Johnson et al., 1991). The underlying mechanism has been debated, some suggesting that we are born with an inherited template that privileges face recognition, others that there is an inborn preference for stimuli that, when fixated, have more elements in the upper fields (two eyes) than in the lower (one mouth; Simion et al., 2002) or that a rapid plasticity that, through intimate contact with the mother, privileges recognition of faces. Whichever explanation turns out to be correct, most would readily accept that the recognition of faces is privileged over the recognition of other objects, especially man-made artifacts such as houses or cars. The general consensus is that face perception is holistic or configural (Rossion et al., 2011) and that distortions of that essential configuration, even a mere inversion, for both faces and bodies (Gliga and Dehaene-Lambertz, 2005) lead to significant difficulties in identifying them and to marked differences in cortical activity (Haxby et al., 2000). Other evidence also points to the robustness of the neural representation for faces. Violations of the basic essential configuration that constitutes a face or body (something that the artist Francis Bacon specialized in) are not readily adapted to, unlike violations of the configuration of man-made objects (Chen and Zeki, 2011). It is evident, then, that there is a significant configuration, departure from which can never qualify a face as beautiful or lead to an aesthetic emotion. Hence it is the way that elements are combined that gives the essential configuration that is critical for recognition of a stimulus as a face (rather than an individual face, which depends upon experience). Only a few outlines are sufficient to qualify a stimulus as being that of a face or a body, as artists from the Ice Age onward and especially artists of the Cycladic civilization recognized. The same is broadly true for bodies, although it appears that the ability to recognize bodies develops somewhat later than for faces (Heron-Delaney et al., 2011). It is worth emphasizing that the areas of the brain that are critical for face recognition can be activated with faces alone, in other words that the faces need not be parts of bodies; equally, the areas of the brain that are critical for the perception of bodies can be activated by headless bodies (Downing et al., 2001). This perhaps emphasizes that a significant configuration that applies to a body does so independently of the face and vice versa, highlighting again the separate treatment of these two entities, although it is also worth emphasizing that the areas of the brain critical for face perception are intimately connected with those critical for body perception (de Gelder, 2006).

Beyond the recognition of a face as a face or a body as a body, there are significant configurations that give it an emotional envelope to qualify it as beautiful or not. It is common knowledge that we are able to classify a face or body from another race or culture as beautiful (Cunningham et al., 1995; Rhodes, 2006) and various characteristics, including youth, symmetry, averageness, and lighter skin colors as well as a combination of large eyes, small noses, and full lips (Jones et al., 1995) have been put forward as playing universal roles in facial attractiveness (for women; Coetzee et al., 2012) although the golden ratio apparently does not (Kiekens et al., 2008; Rossetti et al., 2013). Faces and bodies can thus be said to have a significant configuration, outside of which neither can be qualified as beautiful, which is not the same thing as saying that adherence to that configuration would necessarily qualify a face or a body as beautiful; there clearly are other factors that, in addition to the significant configuration for face or body, arouse the aesthetic emotion. It has been suggested, for example, that rounded body displays and movements are perceived as being warm and friendly (Aronoff et al., 1992); in more general terms, it is common knowledge that certain configurations of the face, the mouth, the eyes, and eyebrows may signal sadness or happiness. Hence the “mysterious laws” that the artist taps are creations that approximate as much as possible to these configurations to arouse the “aesthetic emotion.”

Thus departure from an essential configuration – through inversion or other means – markedly affects the pattern of activity in the brain for faces and bodies while approximation to a significant configuration and therefore to a privileged activation of the sensory areas results in aesthetically more satisfying displays – either of kinetic patterns or of faces (Ishai, 2007) and bodies or of abstract and representational paintings. Once the requirements for a significant configuration are satisfied, there are almost certainly other significant configurations, yet to be discovered, which are critical in qualifying a face as attractive or aesthetic, thus leading (possibly) to an aesthetic emotion. Nor are essential and significant configurations necessarily limited to the attributes I have described above. They may extend into spatial relationships which are also robust, in the sense that impossible relationships appear to be difficult to adapt to neurally, just as violations of the essential configurations of a face are difficult to adapt to neurally (Chen and Zeki, 2011).

One supposes that stimuli with significant configurations are also ones that are more potent in recruiting attentional mechanisms and therefore attracting attention than stimuli that lack them and are therefore more neutral. Theoretically, it should be possible to compare the extent to which stimuli that are experienced as beautiful are more potent in mobilizing the attentional system compared to counterparts that are not. Since significant configurations are, in Bell’s formulation and mine, independent of culture and learning one would expect that the attentional mechanisms would be activated through a bottom-up system. It is of course plausible that the mechanism could be more circuitous and involve feed-back from emotional centers such as the mOFC, an issue that can be addressed experimentally.

## CONCLUSION

The critical points, then, that Bell’s essay brings into experimental focus may be summarized as follows:

(1) By focusing on the “aesthetic emotion” and implying that all works of art are capable of arousing it, his formulation focuses neurobiological attention on what one may loosely call a common path. Implied in this is that there is something common in the emotions aroused by different works that are experienced as aesthetically beautiful, something that finds a correlate in the common activity within the mOFC, a part of the emotional brain whose activity correlates with the experience of beauty. It raises the interesting question of whether, once provoked, the aesthetic emotion can be further differentiated into, for example, visual or musical even if its source and the pathways that lead to the mOFC from the different sensory areas can be so differentiated.
(2) By focusing on a significant form (which I have modified to significant configuration), his formulation raises the question of the difference between aesthetic perception and aesthetic emotion. This leads us to address the question of whether there is some kind of biologically based significant configuration that activates the relevant sensory areas to arouse the “aesthetic perception” and whether it is more potent in mobilizing attentional mechanisms in the brain. What would the pattern of such activation in different domains and for different visual areas be and how would it differ from activation of the same areas by equivalent stimuli which do not lead to an aesthetic percept? Is it maximal activity in the sensory areas, or is it optimal activity, or is it neither but a kind of specific activity in response only to stimuli that have such a configuration? The evidence from the quite voluminous literature on the neurobiology of face perception would seem to favor the principle of significant configuration leading to some kind of privileged activation, as does the neurobiology of body perception. It is surprising that this is also true for the neurobiology of motion perception and, by extension, kinetic art.
(3) Moreover, a privileged activation of an area by a significant configuration is, in Bell’s formulation and mine, independent of culture and upbringing, so one would expect a pretty uniform picture when people from different groupings – whether ethnic or cultural or educational – are studied as indeed seems to be the case with the preferred kinetic stimuli described above. Hence Bell’s formulation focuses attention on what neural mechanisms or significant configurations are common to all humans, irrespective of culture and upbringing and how resistant these configurations are to exposure to different cultures. In simpler terms, is there anything to suggest that neural circuits underlying significant configurations are more robust because more biologically based, and therefore less plastic, than other configurations which are modifiable by exposure to different environments?
(4) A “significant configuration” is an objectively identifiable configuration, with characteristics that can be quantified, as the example of the kinetic stimuli given above, or the relations of the various components of a face to each other that render it attractive, show. Hence, by relating such objective qualities of a stimulus to an activation that leads to an “aesthetic perception,” the long-term aim would be to come to a better understanding of the relationship between objective qualities and the pattern of brain activation that leads to an aesthetic perception and triggers an “aesthetic emotion.” This has of course proved to be difficult even for faces and bodies; it is likely to prove much more difficult for more complex stimuli, such as to be found in paintings.
(5) The filtering of signals to one destination or another: an optimal, or maximal, or specific pattern of activity in an area implies that stimuli which do not have a significant configuration, and hence do not activate the relevant area in the same way, would not lead to an aesthetic perception; they would qualify either as neutral or, if the departure is extreme, as ugly. This has interesting consequences. When humans view a neutral face there is strong activity in the fusiform face area (FFA) but when they view a face that they experience as beautiful there is also a correlated activity in the mOFC. By contrast, when they view a face that is ugly or disfigured, there is, in addition to the activity in the FFA and other areas critical for seeing faces, activity in the amygdala. What neural mechanism is it that determines that signals are channeled to one destination – the mOFC – and not the other? Could it all be based on the pattern of activity provoked by a significant configuration? Or are there other factors besides? This is of course not a problem linked especially to neuroesthetics; it is a critical problem for the whole of cortical neurobiology. Every area of the cerebral cortex has multiple inputs and outputs and whether all the outputs are activated when an area undertakes a given task, or whether outputs are selectively activated depending upon the pattern of activation in that area is a central problem in neurobiology. Neuroesthetics may yet provide fertile ground for addressing this capital issue.

Hence Bell’s aesthetic theory, in somewhat modified form, raises issues that are of importance not only for neuroesthetics and the philosophy of aesthetics but for neurobiology as a whole.

## Conflict of Interest Statement

The authors declare that the research was conducted in the absence of any commercial or financial relationships that could be construed as a potential conflict of interest.

## Footnotes

1 Every day, I attach less importance to intelligence. Every day, I become more aware that it is only outside it that a writer can regain something of our past impressions, that is to say attain something of himself, which is the only material for art”

2 “…touches on very important intellectual problems, indeed perhaps the most important of all for an artist, namely the inferiority of the intellect…and this inferiority, it is after all to the intellect that one appeals to establish it. Because if the intellect does not merit the supreme crown, it alone is capable of discerning it. And if in the hierarchy of instincts it occupies the second rank, it is only intellect that is capable of proclaiming that instinct occupies the first rank”
